# Comparing salt tolerance of beet cultivars and their halophytic ancestor: consequences of domestication and breeding programmes

**DOI:** 10.1093/aobpla/plu083

**Published:** 2014-12-09

**Authors:** Jelte Rozema, Danny Cornelisse, Yuancheng Zhang, Hongxiu Li, Bas Bruning, Diana Katschnig, Rob Broekman, Bin Ji, Peter van Bodegom

**Affiliations:** 1 Systems Ecology, Faculty of Earth and Life Sciences, VU University, De Boelelaan 1085, 1081 HV Amsterdam, The Netherlands; 2 Chang ‘an Agricultural Institute, Dong Ying, Shandong, PR China

**Keywords:** *Beta*, breeding, cultivar, domestication, growth analysis, saline agriculture, salt tolerance, sea beet, sugar beet.

## Abstract

Salt tolerance of higher plants is determined by a complex set of traits, the timing and rate of evolution of which are largely unknown. We compared the salt tolerance of cultivars of sugar beet and their ancestor, sea beet, in hydroponic studies and evaluated whether traditional domestication and more recent breeding have changed salt tolerance of the cultivars relative to their ancestor. Our comparison of salt tolerance of crop cultivars is based on values of the relative growth rate (RGR) of the entire plant at various salinity levels. We found considerable salt tolerance of the sea beet and slightly, but significantly, reduced salt tolerance of the sugar beet cultivars. This indicates that traditional domestication by selection for morphological traits such as leaf size, beet shape and size, enhanced productivity, sugar content and palatability slightly affected salt tolerance of sugar beet cultivars. Salt tolerance among four sugar beet cultivars, three of which have been claimed to be salt tolerant, did not differ. We analysed the components of RGR to understand the mechanism of salt tolerance at the whole-plant level. The growth rate reduction at higher salinity was linked with reduced leaf area at the whole-plant level (leaf area ratio) and at the individual leaf level (specific leaf area). The leaf weight fraction was not affected by increased salinity. On the other hand, succulence and leaf thickness and the net assimilation per unit of leaf area (unit leaf rate) increased in response to salt treatment, thus partially counteracting reduced capture of light by lower leaf area. This compensatory mechanism may form part of the salt tolerance mechanism of sea beet and the four studied sugar beet cultivars. Together, our results indicate that domestication of the halophytic ancestor sea beet slightly reduced salt tolerance and that breeding for improved salt tolerance of sugar beet cultivars has not been effective.

## Abstract (precis)

Our results indicate that the salt tolerance of sugarbeet cultivars is only slightly less than that of their sea beet ancestor and that domestication and selection among sugar beet cultivars have not improved salt tolerance. While the yield of many traditional crops is reduced in salinized soils, sugar beet cultivars are tolerant to increased salinity. It is expected that salt tolerant sugar beet will be productive under seawater and brackish water irrigation in saline agriculture. The use of brackish and saline water for saline agriculture helps to prevent depletion of fresh water on earth.

## Introduction

The availability of fresh water for use in agriculture is becoming increasingly limited. In contrast, the availability of brackish and more saline water is practically unlimited. If brackish water and seawater could be used for crop cultivation on salinizing soils, vast amounts of fresh water would be saved (Rozema and Flowers 2008). Saline agriculture that exploits brackish water and salinized soils can deliver not only food products for human consumption, such as vegetables and fruits, but also cattle fodder, raw materials for industrial use, biofuel and biodiesel.

Saline agriculture is in need of salt-tolerant crops (Munns *et al.* 2012). Salt tolerance of crops has been evaluated by Maas and Hoffman (1977) and Maas (1985) using threshold and slope values of the crop yield response to increased salinity as criteria. Crop salt tolerance can be considerable, but is generally much less than that of native plant species, halophytes, from salt marshes and saline inland sites (Rozema and Flowers 2008; Rozema and Schat 2013). Among higher plants, salt tolerance evolved independently and repeatedly (Flowers *et al.* 2010; Bennett *et al.* 2013). However, the timing and rate of evolution of salt tolerance are largely unknown. Recent molecular genetic analyses have revealed that salt tolerance among Salicornioideae and Chenopodioideae, to which beet belongs, evolved some 30 Mya (Kadereit *et al.* 2006; see Rozema and Schat 2013). Such salt tolerance is a multigenic complex of traits interacting at the whole plant, tissue, cellular and molecular level (Tester and Davenport 2003; Munns and Tester 2008; Shabala and Mackay 2011). One consequence of this evolutionary complexity may be that evolution of salt tolerance is slow and may take place gradually or stepwise, driven by high salinity of the environment. This is reflected by the existence of a continuum of degrees of salt tolerance among higher plants, gradually changing from salt sensitive to highly salt-tolerant plant species (Flowers *et al.* 2010).

One way to obtain salt tolerance in crops is to domesticate halophytes. Beet is such an example, where the salt tolerance relates to its ancestor, sea beet. Edible beets such as fodder beet, table beet, red beet and sugar beet have been derived from sea beet, its coastal ancestor, through the process of domestication over thousands of years (Zohary and Hopf 2000; Biancardi *et al.* 2012). Since about 1800, selection and breeding for more salt-tolerant cultivars of sugar beet has been taking place.

The salt tolerance of plants and crops may be assessed by comparing plant growth rate under saline and non-saline conditions (Shannon and Grieve 1999). By comparing the relative growth rate (RGR), rather than the absolute growth of plants, in response to increased salinity, the estimated salt tolerance will be less dependent on the length of the growth period (Rozema and Schat 2013). The RGR of sea beet and sugar beet in response to salinity has been assessed in a number of reports (Marschner *et al.* 1981; Rozema *et al.* 1993; Koyro 2000; Niazi *et al.* 2000; Ghoulam and Fares 2001; Ghoulam *et al.* 2002; Shaw *et al.* 2002; Niazi 2007; Daoud *et al.* 2008; Hajiboland *et al.* 2009; Wu *et al.* 2013), but other growth components needed for growth analysis (as in Rozema *et al.* 1993 and Niazi *et al.* 2000) were not included. For example, Ghoulam *et al.* (2002) reported that increased external NaCl concentrations caused a great reduction in growth parameters such as leaf area, and fresh and dry weight of leaves and roots, but that leaf number was less affected. Ghoulam *et al.* (2002) assumed that all plant parameters measured could be used as indicators of salt tolerance. Values of some growth parameters and components of RGR (e.g. shoot and root biomass, leaf area, leaf thickness and ratios of these in response to salinity) do not always represent reliable indicators of salt tolerance since they may respond to salinity indirectly. The value and novelty of growth analysis and its components as we report here lies in the combined analysis of many interacting plant traits in response to increased salinity, providing new insights into the understanding of adaptations to salinity at the whole-plant level.

In this paper, we address three research questions: (i) Has the process of domestication from sea beet to sugar beet changed the salt tolerance? (ii) Is the salt tolerance of sugar beet cultivars, selected for higher salt tolerance, indeed higher in comparison with control or salt-sensitive sugar beet cultivars? (iii) Do components of growth in response to salinity contribute to understand salt -tolerance mechanisms in sea beet and sugar beet at the whole-plant level? To assess salt tolerance, we compared the RGR of sea beets and sugar beets at six salinity levels. For the third research question, we examined RGR, its components as well as leaf thickness and succulence in response to salinity.

## Methods

### Sea beet and sugar beet cultivars

The natural geographical distribution of sea beet (*Beta vulgaris* ssp. *maritima* (van der Meijden 2005), hereafter *Beta maritima* or sea beet) ranges from the Mediterranean to the Atlantic coastline (Rozema 1996; Biancardi *et al.* 2012; den virtuellen flora). Sea beet naturally occurs at the upper fringes of salt marshes, which are only occasionally flooded by seawater. The salt tolerance of sea beet might relate to hypersaline soil conditions occurring during the summer period, when soil salinity in the surface soil of the upper marsh is high (Waisel 1972; Rozema *et al.* 1987; Rozema 1996). Sea beet is a short to longer lived perennial species, while the sugar beet (*B. vulgaris* ssp. *vulgaris*.) is predominantly biennial. For the nomenclature of sea beet and sugar beet, we follow van der Meijden (2005).

Seeds of sea beet were obtained from Dr Arjen de Vos Zilt Proefbedrijf, Den Burg, Texel, The Netherlands, collected during the autumn of 2012 from saline coastal sites on the Island of Texel. Before sowing, seeds were stored in a dry and cold room (4–8 °C) for 4 months. Seeds of cultivars of sugar beet were obtained from commercial sources (unnamed here for reasons of commercial confidentiality); three cultivars were claimed to be salt tolerant, i.e. cultivar S06M38285 (coded in this paper as ST1), cultivar 847911 (ST2) and cultivar S10R43463 (ST3), and cultivar S11R38516 (SS1) was claimed to be salt sensitive.

### Greenhouse conditions

The effect of increased salinity on growth parameters of four sugar beet cultivars and sea beet was studied in an experiment in a climate-controlled greenhouse using hydroponic culture. Seeds were sown on peat soil in plastic trays (seed pot soil Jongkind, Aalsmeer, The Netherlands) at the beginning of June 2013. Photosynthetic active radiation (PAR) levels in the greenhouse from natural sunlight and additional lamps were, on average, 250 μmol m^−2^ s^−1^. The temperature was 21/16 °C (day/night) and the relative humidity of air varied between 70 % (8 h day) and 90 % (16 h night). After 9 days, germination and emergence of the sugar beet cultivars were close to 100 %, while ∼40 % of the sea beet had germinated and emerged. This experiment was repeated three times (winter, spring, autumn with differences in the PAR in the greenhouse) with the same overall results: we have chosen to present the results of the experiment carried out in June 2013.

### Hydroponic cultivation with increased salinity

Seedlings of the same height (∼5 cm) were carefully washed free of soil with demineralized water and transplanted to hydroponic culture in 5 L plastic trays filled with modified ¼-strength Hoagland solution, containing (in mM): K^+^, 3.001; Ca^2+^, 2; Mg^2+^, 0.5; $N{O_{3}}^{-}$, 5; $N{H_{4}}^{+}$, 1.001; $\mathrm{HP}O_{4}{}^{2-}$, 1; $SO_{4}{}^{2-}$, 0.516; Cl^−^, 0.001; $H_{2}B{O_{3}}^{-}$, 0.025; Mn^2+^, 0.002; Zn^2+^, 0.002; Cu^2+^, 0.001; Mo^2+^, 0.001; Fe-Na-EDTA, 0.01, buffered with 2 mM 2(*N*-morpholino)ethanesulfonic acid, pH 6.0. This solution was continuously aerated. Individual seedlings, wrapped in nylon cotton plugs, were placed in holes in a foam plate floating on the nutrient solution. There were five rows of five holes in each plate. Sea beet and the four sugar beet cultivars were randomly assigned to one of the five rows. The seedlings were allowed 6 days to recover from transplanting. Thereafter, a daily stepwise increase of 5 dS m^−1^ salinity was applied (electrical conductivity (EC) ∼50 mM NaCl) by using a 5-M NaCl stock solution until the desired EC value was reached. This was applied to avoid an osmotic shock and continued until six salinity levels were realized (0.4, 5, 10, 15, 20 and 30 dS m^−1^; ∼0.4, 50, 100, 150, 200 and 300 mM NaCl; 300 mM NaCl represents ∼66 % of the salinity of seawater). The EC value 0.4 dS m^−1^ of the control salinity was due to the nutrients contained in the solution. Each salinity level was replicated six times; in total there were 36 plastic trays. The nutrient solutions were renewed every week. Salinity levels were checked with a WTW hand-held EC meter (Cond 3110, WTW GmbH, Weilheim, Germany). The position of the trays with seedlings was re-randomized daily, except weekends, to avoid site effects.

### Growth analysis

Two plants of sea beet and the sugar beet cultivars were harvested from each tray 3 days after reaching the final salinity level (first harvest). The remaining three plants of sea beet or cultivar from each tray were harvested 10 days later (final harvest). Average values of these plants from each tray were used for plant growth analysis, using only the true replicates (trays) for analysis. At harvest, plants were rinsed with demineralized water and carefully blotted dry, separated into roots and leaves for fresh weight measurement. For leaf area ratio (LAR), the leaf area of the cotyledons, first, second and third leaf pairs were measured with an LI-COR 3100 leaf area meter (Li-COR, Inc., Lincoln, NE, USA). For succulence, the leaf area of the first (and oldest) leaf pairs was measured with an LI-COR 3100 leaf area meter. Leaf thickness of the first (and oldest) leaf pairs, except the cotyledons, was measured with a thickness gauge (no 2046-08, accuracy 0.01 mm, Mitutoyo, Japan) avoiding the leaf veins. The average of both leaves of a leaf pair was used. Dry weight was determined from oven-dried plant material (70 °C, 48 h).

### Calculation of RGR and other growth parameters

To calculate relative growth rate (RGR) and its components specific leaf area (SLA), leaf area ratio (LAR), leaf weight fraction (LWF; sometimes referred to as leaf mass ratio or fraction, LMR or LMF) and unit leaf rate (ULR) for the sugar beet cultivars and sea beet we used the following equation (Hunt *et al.* 2002; Poorter 2002; Poorter and Garnier 2007; Poorter *et al.* 2012):

(1) $$\mathrm{RGR}=\mathrm{ULR}\times \mathrm{LWF}\times \mathrm{SLA}=\frac{\ln(w_{2})-\ln(w_{1})}{t_{2}-t_{1}}$$

where *w*_2_ and *w*_1_ represent the total plant weight for the final and the first harvest, respectively. *t*_2_−*t*_1_ is the time difference in days between the first and the second harvest. To calculate ULR, LAR, LWF and SLA for the sugar beet cultivars and sea beet, the following equations were used:

(2) $$\mathrm{ULR}=\frac{\left(w_{2}-w_{1})\times (\ln(L_{A2})-\ln(L_{A1})\right)}{\Delta L_{A}\times (t_{2}-t_{1})}$$

(3) $$\mathrm{LAR}=\frac{L_{A}}{w}$$

(4) $$\mathrm{LWF}=\frac{L_{\mathrm{DW}}}{w}$$

(5) $$\mathrm{SLA}=\frac{L_{A}}{L_{\mathrm{DW}}}$$

where *L*_A_ is the total leaf area (in cm^2^), excluding cotyledons, Δ*L*_A_ is the difference in total leaf area between the first and the final harvest and *L*_DW_ is the total leaf dry weight (in g). The product of SLA and LWF equals LAR. Leaf succulence was calculated as follows:

(6) $$\mathrm{Succulence}=\frac{L_{\mathrm{FW}}}{L_{A}}$$

where *L*_FW_ is the total leaf fresh weight (in g), excluding cotyledons.

Any significant difference in RGR or its components does not show to what extent a growth parameter changed relative to the control at any particular salinity. The values of the ‘relative effect’ provide additional information on the strength of the response. This relative effect was calculated by expressing the change of each growth parameter in response to the five increasing salinity levels as a percentage of their values in the absence of salinity (at 0.4 dS m^−1^; Table 1A–G).

**Table 1.** (A–G) The ‘relative effect’ of the effects of increased salinity on the growth parameters RGR, LAR, SLA, LWF, leaf succulence, leaf thickness and ULR expressed as percentage relative to EC 0.4 dS m^−1^ (100 %) for the four individual sugar beet cultivars as well as for the four cultivars together (‘all cultivars’) and sea beet. SS1 = salt-sensitive sugar beet; ST1–3 = salt-tolerant sugar beet cultivars.

	Salinity EC (dS m^−1^)	0.4	5	10	15	20	30
(A) RGR in response to increased salinity, as % relative to EC 0.4 dS m^−1^	Cultivar SS1	100	127	122	101	90	63
	Cultivar ST1	100	125	98	113	80	42
	Cultivar ST2	100	107	111	97	79	49
	Cultivar ST3	100	125	116	99	75	54
	All cultivars	100	121	112	102	81	53
	Sea beet SB	100	112	135	112	102	89
(B) LAR in response to increased salinity, as % relative to EC 0.4 dS m^−1^	Cultivar SS1	100	92	70	68	56	41
	Cultivar ST1	100	122	85	73	47	48
	Cultivar ST2	100	99	69	83	62	60
	Cultivar ST3	100	82	73	75	61	48
	All cultivars	100	100	74	74	56	49
	Sea beet	100	86	79	66	68	54
(C) SLA in response to increased salinity, as % relative to EC 0.4 dS m^−1^	Cultivar SS1	100	105	79	70	60	51
	Cultivar ST1	100	115	87	70	49	50
	Cultivar ST2	100	111	79	80	65	62
	Cultivar ST3	100	89	76	73	60	54
	All cultivars	100	105	80	73	58	54
	Sea beet	100	117	108	91	97	57
(D) LWR in response to increased salinity, as % relative to EC 0.4 dS m^−1^	Cultivar SS1	100	107	102	103	108	121
	Cultivar ST1	100	113	97	96	93	98
	Cultivar ST2	100	99	93	92	98	101
	Cultivar ST3	100	100	100	101	98	107
	All cultivars	100	104	98	98	99	107
	Sea beet	100	91	92	94	113	93
(E) ULR in response to increased salinity, as % relative to EC 0.4 dS m^−1^	Cultivar SS1	100	117	146	193	206	197
	Cultivar ST1	100	105	92	280	213	136
	Cultivar ST2	100	94	131	176	175	126
	Cultivar ST3	100	127	137	167	185	137
	All cultivars	100	110	127	202	194	146
	Sea beet	100	88	104	127	116	150
(F) Succulence in response to increased salinity, as % relative to EC 0.4 dS m^−1^	Cultivar SS1	100	115	124	145	150	167
	Cultivar ST1	100	96	114	139	179	119
	Cultivar ST2	100	122	138	141	136	154
	Cultivar ST3	100	136	141	140	140	177
	All cultivars	100	116	130	141	153	153
	Sea beet	100	129	130	157	147	146
(G) Leaf thickness in response to increased salinity, as % relative to EC 0.4 dS m^−1^	Cultivar SS1	100	129	148	193	214	225
	Cultivar ST1	100	128	124	167	175	161
	Cultivar ST2	100	122	130	161	166	173
	Cultivar ST3	100	136	140	179	192	209
	All cultivars	100	128	135	175	186	191
	Sea beet	100	144	152	179	176	179

### Statistical analyses

To evaluate whether salt tolerance (i.e. the RGR response to increased salinity) differed among the cultivars and sea beet, we executed a two-way ANOVA on RGR with salinity levels (0, 5, 10, 15 20 and 30 ds m^−1^) and beet cultivar (SS1, ST1, ST3, ST2 and SB) as fixed factors and the salinity × beet cultivar interaction term calculated. Because the salinity × cultivar interaction term was significant, we used Tukey HSD multiple comparison post-hoc tests to compare all unique 30 treatment combinations of RGR, salt and cultivar.

**Figure 1.**
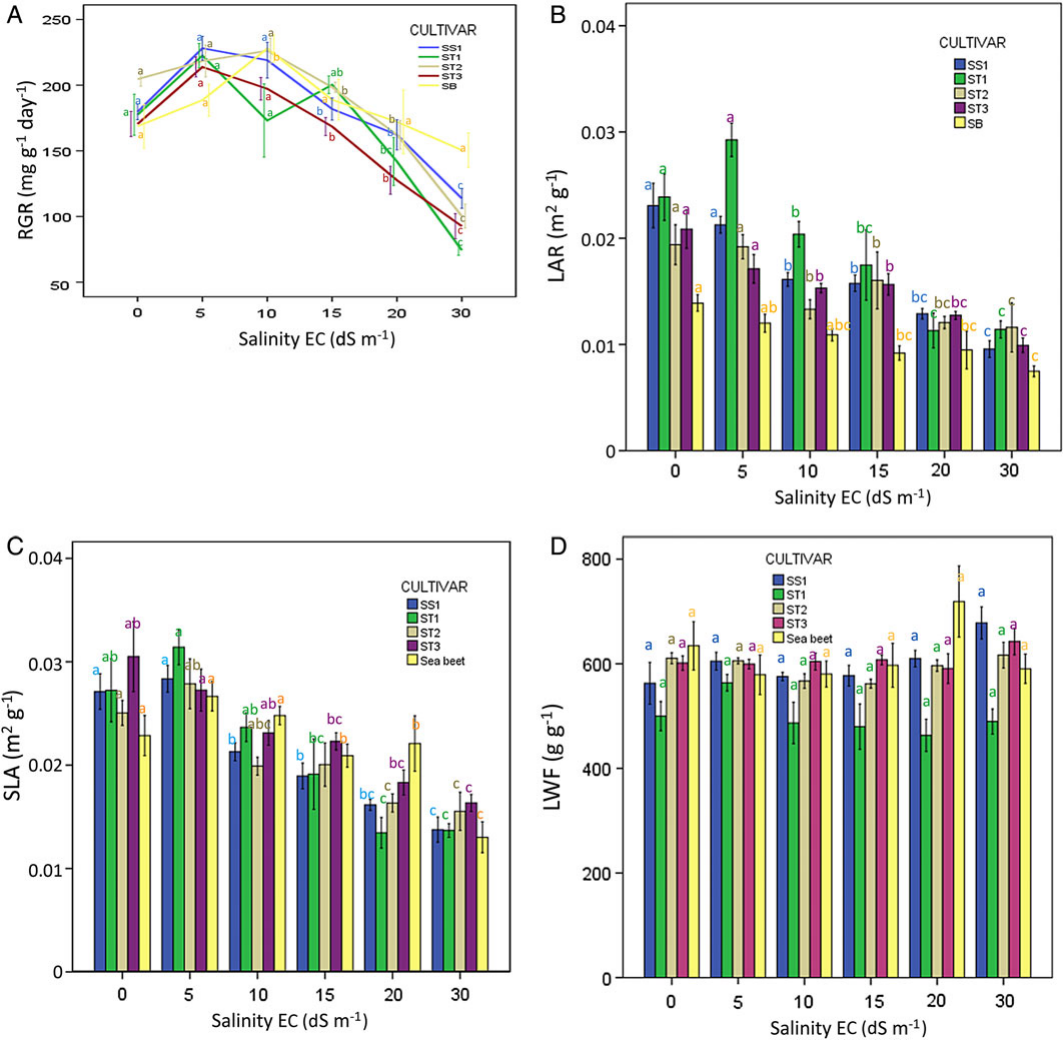

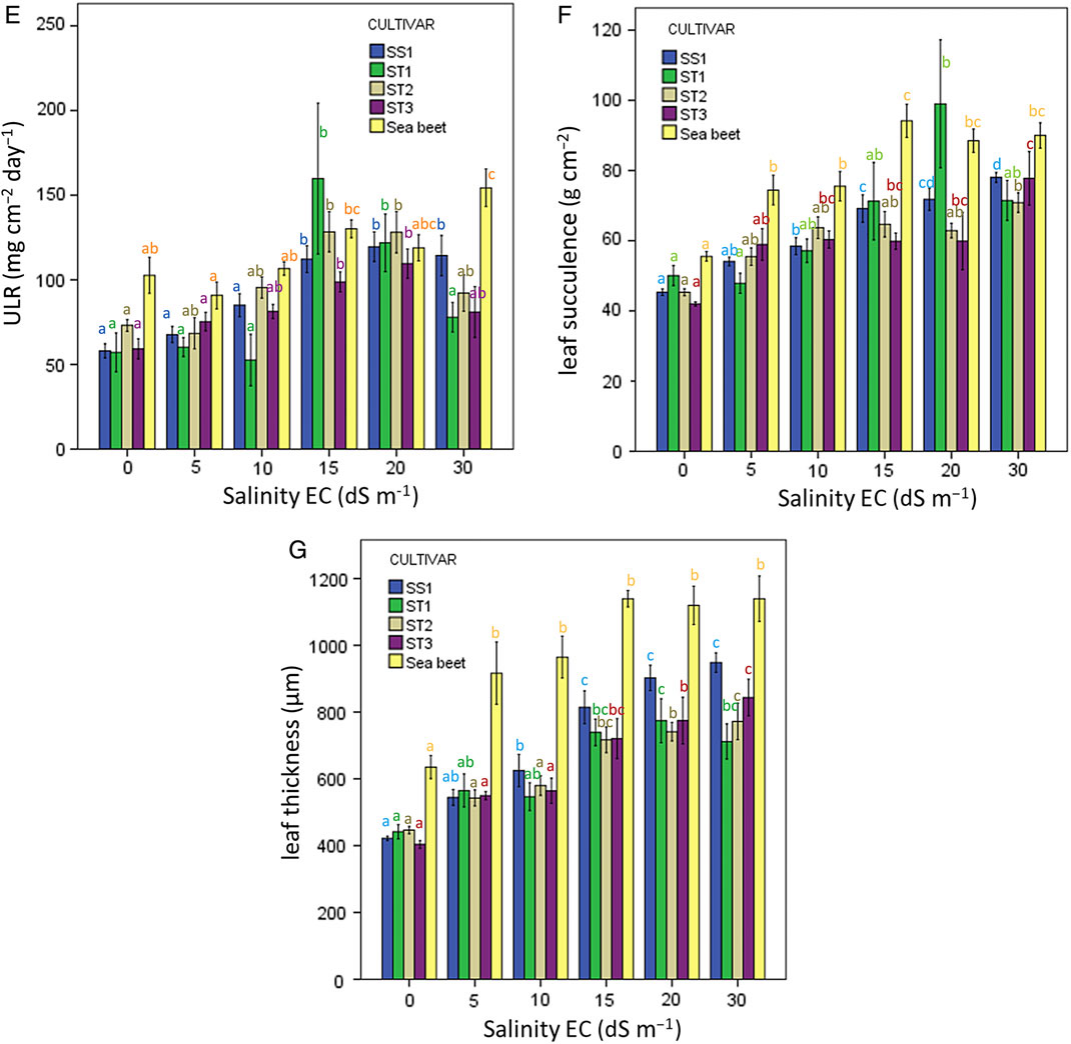
(A–G) Relative growth rate and its components in response to increasing salinity 0, 5, 10, 15, 20, 30 EC values dS m^−1^ (10 dS m^−1^, about 100 mM NaCl) in hydroponic culture. The average values with standard error of the mean of RGR (mg g^−1^ day^−1^) (A); LAR (m^2^ g^−1^) (B), SLA (m^2^ g^−1^) (C); LWF (g g^−1^) (D); leaf succulence (g cm^−2^) (F); leaf thickness (μm) (G); ULR (mg cm^−2^ day^−1^) (E) of sugar beet cultivars SS1 (considered salt sensitive), ST1, ST2, ST3 considered to be salt tolerant and sea beet (SB). The figures contain the results of one-way ANOVAs testing the effect of increasing salinity on individual growth parameters based on Tukey's HSD post-hoc tests. Different letters indicate a significant difference at *P*< 0.05.

To understand better the ecophysiological mechanisms of salt tolerance of sugar beet and sea beet, the plant growth traits RGR, LAR, SLA, ULR, LWF, leaf succulence and leaf thickness were analysed in response to salinity. Given that in this case, we were interested in ecophysiological responses of these traits of each individual beet variety, we used one-way ANOVAs with the salinity level as a fixed factor followed by Tukey's post-hoc HSD tests for each individual cultivar and sea beet separately (Fig. 1A–G). Prior to analysis, normality and homogeneity assumptions were checked by visual inspection of the residuals for undesired patterns and by Levene's test for homogeneity of variances. All statistical tests were performed using SPSS 21.0.

## Results

### RGR in response to increased salinity and comparison of sugar beet cultivars and sea beet

A two-way ANOVA was used to determine the effects of salt treatment and cultivar on RGR. All factors were found to be statistically significant (cultivar *F*_4,148_ = 7.1; *P* < 0.001; salt *F*_5,148_ = 47.2; *P* < 0.001; cultivar × salt *F*_20,148_ = 2.8; *P* < 0.001). Tukey's HSD multiple comparison *post-hoc* tests were used to detect the significance of effects of salinity × cultivar combinations on RGR. We found no significant difference in the RGR response to increased salinity among the salt-sensitive (SS1) and salt-tolerant (ST1, ST2, ST3) sugar beet cultivars. Of the data set containing all combinations of RGR values of sea beet and the four cultivars of sugar beet and six salinity levels, only at 30 dS m^−1^ the RGR of sea beet was found to be significantly larger than that of the sugar beet cultivars. A possible trade-off between this larger salt tolerance of sea beet in comparison with sugar beet cultivars and growth rate was tested, and it was found that RGR values of sea beet and sugar beet cultivars at 0.4 dS m^−1^ did not significantly differ.

When sugar beet cultivars and sea beet were analysed individually for their response to salinity, the RGR of sea beet appeared to increase at 10 dS m^−1^ (*F*_5,23_ = 3.0; *P* = 0.03) compared with that at 0.4 dS m^−1^ (Fig. 1A), but was not reduced until the highest salinity level (30 dS m^−1^). In contrast to sea beet, the RGR of the four sugar beet cultivars reduced with increased salinity (one-way ANOVA for individual cultivars, SS1, *F*_5,30_ = 27.2; *P* < 0.001; ST1, *F*_5,30_ = 18.1; ST2, *F*_5,30_ = 25.2; *P* < 0.001; ST3, *F*_5,30_ = 27.2; *P* < 0.001, outcome of Tukey's *post-hoc* HSD tests, Fig. 1A), most pronounced at 20 and 30 dS m^−1^.

When pooling the RGR values of various sugar beet cultivars to get the RGR of ‘all cultivars’, it was shown that the RGR at 20 and 30 dS m^−1^ declined significantly (compared with lower salt conditions and compared with one another) to 81 and 53 % of the value at 0.4 dS m^−1^, respectively. For sea beet, on the other hand, the RGR at 20 dS m^−1^ was 102 % and at 30 dS m^−1^ was 89 % compared with that at 0.4 dS m^−1^ (Table 1A). Although not statistically significant for the sea beet and sugar beet cultivars separately (Fig. 1A), the RGR of ‘all cultivars' in response to EC salinity at 5 dS m^−1^ was significantly higher than that at 0.4 dS m^−1^ (*F*_5,165_ = 48.8; *P* < 0.001 including sea beet; *F*_5,136_ = 57.9, *P* < 0.001 when excluding sea beet).

### LAR and SLA in response to salinity

Leaf area per plant biomass (LAR) and leaf area per leaf biomass (SLA) of all individual sugar beet cultivars and sea beet were significantly reduced [for LAR (SS1, *F*_5,30_ = 22.1; ST1, *F*_5,30_ = 12.9; ST2, *F*_5,30_ = 12.1; ST3, *F*_5,30_ = 3.9; SB, *F*_5,23_ = 6.9, respectively; all *P*-values <0.001). For SLA (SS1, *F*_5,30_ = 23.6; ST1, *F*_5,30_ = 11.3; ST2, *F*_5,30_ = 8.5; ST3, *F*_5,30_ = 8.5; SB, *F*_5,23_ = 8.9 respectively; all *P*-values <0.001)] with increased salinity (Fig. 1B and C). At 10 dS m^−1^, the LAR of ‘all cultivars’ decreased to 75 % and of sea beet to 79 % compared with  that at 0.4 dS m^−1^; at 30 dS m^−1^, LAR was reduced to 49 and 54 %, for sugar and sea beet, respectively. Specific leaf area at 10 dS m^−1^ decreased to 80 % for ‘all cultivars' and to 54 % at 30 dS m^−1^, but for sea beet at 10 dS m^−1^ SLA increased to 107 % and only reduced to 57 % at 30 dS m^−1^ (Table 1B–G). At high salinity (30 dS m^−1^), both leaf area per plant biomass (LAR) and leaf area per leaf biomass (SLA) of all sugar beet cultivars and sea beet were reduced by ∼50 % when compared with that at 0.4 dS m^−1^ (Table 1B and C).

### LWF, leaf succulence, leaf thickness and ULR in response to salinity

Leaf weight fraction was not significantly affected by increased salinity for any of the four sugar beet cultivars tested (SS1, *F*_5,30_ = 1.9; *P* = 0.113; ST1, *F*_5,30_ = 1.2, *P* = 0.329; ST2, *F*_5,30_ = 0.9, *P* = 0.471; ST3, *F*_5,30_ = 2.8; *P* = 0.360 respectively), nor for sea beet (SB, *F*_5,23_ = 1.5; *P* = 0.218, Fig. 1D). However, leaf succulence (SS1, *F*_5,30_ = 26.8; ST1, *F*_5,30_ = 6.8; ST2, *F*_5,30_ = 6.4; ST3, *F*_5,30_ = 3.7; SB, *F*_5,30_ = 10.8, respectively; all *P*-values <0.001) and leaf thickness (SS1, *F*_5,30_ = 34.7; ST1, *F*_5,30_ = 7.7; ST2, *F*_5,30_ = 12.4; ST3, *F*_5,30_ = 14.7; SB, *F*_5,30_ = 10.3 respectively; all *P*-values <0.001) significantly increased with increased salinity (Fig. 1F and G)—∼50 % increase in leaf succulence from 15 to 30 dS m^−1^ for both ‘all cultivars' and sea beet and 70–90 % for leaf thickness for ‘all cultivars' and sea beet. Absolute leaf thickness of sea beet was ∼50 % higher than that of ‘all cultivars' at 0.4 dS m^−1^ and exceeded that of ‘all cultivars' substantially at all salinity levels (Fig. 1F). Unit leaf rate increased significantly under raised salinity (Fig. 1G, SS1, *F*_5,30_ = 12.2, *P* < 0.001; ST1, *F*_5,30_ = 4.1, *P* = 0.006; ST2, *F*_5,30_ = 4.5, *P* = 0.003; ST3, *F*_5,30_ = 7.5; *P* < 0.001; SB, *F*_5,23_ = 7.8, *P* < 0.001 for the sugar beet cultivars and for sea beet, respectively). At 10, 15 and 20 dS m^−1^ ULR of ‘all cultivars' increased to 127, 202 and 194 %, respectively, while at 30 dS m^−1^ ULR of both ‘all cultivars' and sea beet was ∼150 % in comparison with 0.4 dS m^−1^.

## Discussion

### Domestication and breeding affect salt tolerance?

In this paper, we question whether salt tolerance of beet cultivars derived by domestication from their coastal ancestor, sea beet, was reduced since beet cultivars are often cultivated in less saline environments than in the coastal, maritime habitat of sea beet. From the results presented in Fig. 1A, we inferred that RGR values of sea beet exceed that of the four sugar beet cultivars tested only at the highest salinity level applied (i.e. 30 dS m^−1^). This indicates that salt tolerance of sea beet is slightly higher than that of the four sugar beet cultivars tested. This might indicate that during the process of domestication from sea beet to sugar beet, salt tolerance of cultivars has reduced slightly, but not to a large extent.

Given the finding of some reduced salt tolerance of sugar beet cultivars compared with their sea beet ancestor, it may be questioned if there has been a trade-off between salt tolerance and growth rate. In the literature the metabolic and energetic costs of increased stress tolerance, including salt tolerance, have been associated with a reduced RGR, which is apparent under non-stressed, i.e. non-saline conditions (Lambers and Poorter 1992; Poorter and Garnier 2007). Energetic costs of increased salt tolerance may, for example, relate to the energy required for ion compartmentation, ion transport systems and synthesis of compatible osmolytes such as proline and glycine betaine (Munns and Tester 2008). We compared the RGR values of the four sugar beet cultivars and sea beet at 0.4 dS m^−1^ and found no significant difference. We found therefore no experimental evidence supporting the hypothesis of a trade-off between salt tolerance and growth rate: the slightly higher salt tolerance of sea beet is not associated with a reduced growth rate.

Remarkably, the relative values in Fig. 1A indicated that the RGR of sea beet and all cultivars at 5 (and 10 dS m^−1^) tend to be higher than that at 0.4 dS m^−1^. The RGR of sea beet at 10 dS m^−1^ was higher than that at 0.4 dS m^−1^; analysis of pooled RGR data (‘all cultivars') for sugar beet demonstrated an increased RGR at 5 dS m^−1^ when compared with that at 0.4 dS m^−1^. Similarly increased growth rate of the sea beet under ‘mild salinity’ (EC 5.5 dS m^−1^ in Hajiboland *et al.* (2009)) when compared with the control treatment has been reported in field studies by Goh and Magat (1989), Almodares and Sharif (2005), Sepaskhah *et al.* (2006), Hajiboland *et al.* (2009) and Szulc *et al.* (2010). The detailed physiological background of such NaCl-stimulated growth remains unclear (Flowers and Colmer 2008; Rozema and Flowers 2008; Katschnig *et al.* 2013, 2014; Rozema and Schat 2013).

### Breeding for improved salt-tolerant sugar beet cultivars

Domestication of edible beet cultivars from sea beet took thousands of years. More recently, since about 1800 (Zohary and Hopf 2000; Biancardi *et al.* 2012), with growing economic interest in sugar for human consumption and industrial purposes, sugar beet cultivars have been selected for large beet size and weight and a high sucrose content. This sucrose may be converted into ethanol and there is the perspective of obtaining biofuel (Asif and Muneer 2007) by cultivation of sugar beet cultivars with improved salt tolerance on salinized land (Yuan *et al.* 2008; Balat and Balat 2009; Zhang *et al.* 2011; Liu *et al.* 2012). Therefore it becomes of interest to seed breeding companies to develop sugar beet cultivars with improved salt tolerance. We obtained three cultivars claimed to have such improved salt tolerance and compared their tolerance with a supposedly salt-sensitive cultivar. However, no significant differences were found among the four sugar beet cultivars tested, indicating that salt tolerance of assumed salt-tolerant sugar beet cultivars ST1, ST2 and ST3 as indicated by a seed breeding company, did not differ significantly from the salt tolerance of the declared salt-sensitive cultivar SS1. Our findings are based on a short-term (∼2 weeks) hydroponic study. We cannot rule out, however, that salt-tolerance tests of a field-based study would show results different from hydroponic studies.

More generally, lack of success in traditional breeding and selection for improved salt tolerance of some crops has been discussed and questioned by Shannon and Grieve (1999), Flowers (2004), Flowers and Flowers (2005), Munns (2005), Colmer *et al.* (2005), Munns and Tester (2008) and Ashraf and Akram (2009). One explanation for our findings of unsuccessful breeding and selection for increased salt tolerance of sugar beet cultivars is that traditional breeding and selection have not altered key traits involved in the multigenic and complex mechanism of salt tolerance (Flowers and Yeo 1995; Munns 2005; Munns and Tester 2008). Another explanation has been a lack of genetic variation for salt tolerance among the cultivars of, for example, rice (*Oryza sativa*) (Flowers 2004; Flowers and Flowers 2005). Alternatively, lack of success might relate to the use of unreliable indicators of salt tolerance. We have argued that we regard the RGR of the entire plant a reliable plant trait to quantify salt tolerance, being independent of the duration of an experiment. Changes of other growth parameters and components of RGR (e.g. shoot and root length and biomass, leaf area, leaf thickness and ratios of these) in response to salinity do not always represent reliable indicators of salt tolerance. For example, reduced leaf area should not necessarily be seen as a salt sensitivity symptom but may also indicate increased succulence. In addition, physiological and metabolic traits such as shoot Na^+^ or proline concentration can be unreliable salt-tolerance indicators (Wu *et al.* 2013).

### Hydroponic and field studies of crop salt tolerance

Across the salinity levels used in our hydroponic study, RGR of the four sugar beet cultivars tested was significantly reduced by ∼20 % at 15–20 dS m^−1^ and to ∼50 % at 30 dS m^−1^, while RGR of sea beet remained almost unaffected up to 30 dS m^−1^. This roughly agrees with earlier hydroponic and field studies on sea beet, sugar beet and fodder beet cultivars (Marschner *et al.* 1981; Rozema *et al.* 1993; Koyro 2000; Niazi *et al.* 2000; Ghoulam and Fares 2001; Ghoulam *et al.* 2002; Niazi 2007; Daoud *et al.* 2008; Hajiboland *et al.* 2009; Zare *et al.* 2012; Wu *et al.* 2013). Salinity and nutrient levels, temperature and light conditions can be well controlled in hydroponic experiments in climate-controlled growth chambers and greenhouses, while there may be considerable spatial variation in soil salinity in the field, which is also affected by temperature, precipitation and evapotranspiration.

Salt tolerance of crops in the field has been assessed by relating the salinity of irrigation water or soil salinity to crop yield, and deriving threshold and slope values from crop yield–salinity relationships (Maas and Hoffman 1977; Maas 1985). Our hydroponic study indicates that sugar beet RGR at 30 dS m^−1^ is still ∼50 % of that of the non-saline treatment (Table 1A). This 30 dS m^−1^ salinity is much higher than the EC 7 dS m^−1^ threshold value for sugar beet yield in the field according to Maas and Hoffman (1977) and Maas (1985), and also higher than the recently proposed ST index, indicating a value for sugar beet of EC 16.4 dS m^−1^ (Steppuhn *et al.* 2005*a*, *b*), proposed as an indicator of the inherent salinity tolerance or resistance of agricultural crops. The ST index is mainly composed of the EC value of the root zone with 50 % reduction in crop yield (C_50_) relative to the non-saline yield. EC values of the C_50_ salt-tolerance index always represent larger values than threshold EC values, since the latter are derived from an earlier part of a response curve. A C_50_ value as a measure of crop salt tolerance may also be applied to hydroponic studies since C_50_ values can be inferred from growth salinity response curves (and seems to be ∼30 dS m^−1^ for sugar beet according to our study). While we assessed salt tolerance using biomass increase of below-ground and above-ground plant parts during a time interval, in agricultural studies crop yield obtained under saline and non-saline field conditions is usually compared. However, salt tolerance inferred from the yield of, for example, cereal crops (where yield follows flowering) may have a different physiological interpretation than that of crop yields where other plant parts (root or above-ground shoot biomass) are harvested. Care must thus be taken when comparing salt tolerances based on yields.

In contrast to growth of mature plants, halophyte seed germination may be relatively salt sensitive as germination tends to occur during seasonal lows of soil surface salinity. After emergence, seedlings need to survive in saline environments (Waisel 1972; Rozema 1975; Ungar 1978). In the case of sugar beet, EC soil salinity values exceeding 3 dS m^−1^ reduce germination and emergence (Steppuhn *et al.* 2005*b*). Therefore, we do not rule out that salt tolerance of adult sugar beet plants may differ from seedlings. However, while salt-tolerance measurements of longer duration in the field may be more useful to the agronomist, comparing salt tolerances of seedlings in hydroponics in a climate-controlled greenhouse is reliable, reproducible and can accommodate large numbers of genotypes over relatively short times. We recommend hydroponic studies be part of any screening programme, which should include field studies for selected genotypes.

### Growth parameters in response to salinity

Our hydroponic study allowed us to assess the effect of increasing salinity on various growth parameters under controlled environmental conditions. From a whole-plant point of view, this may help to determine how sugar beet and sea beet plants adapt to increased salinity. Along with RGR, LAR and SLA decreased substantially with increased salinity; LWF remained unchanged, while the increase of leaf succulence and leaf thickness was pronounced (50–90 % increase at 30 dS m^−1^ compared with 0.4 dS m^−1^).

The growth rate reduction of the sugar beet cultivars with increased salinity thus is mainly associated with reduced leaf area, both at the whole-plant level (LAR) and that of the individual plant leaf (SLA), together with increased leaf thickness and succulence. This could be explained by reduced cell expansion due to reduced water uptake from a saline root environment (Rozema *et al.* 1987; Rozema 1991; Munns and Tester 2008; Shabala and Mackay 2011). The reduced growth rate of beet by salinity may be ascribed to the osmotic component of salt treatment (Rozema *et al.* 1987; Munns and Tester 2008).

**Figure 2.**
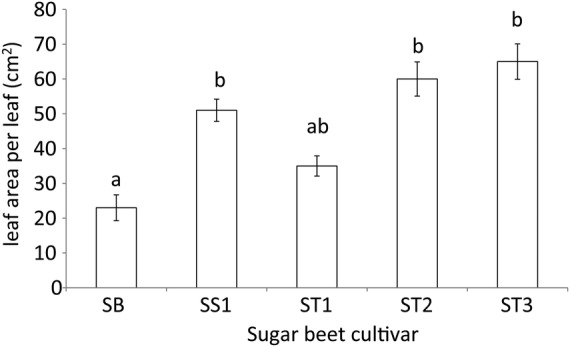
Average leaf area (cm^2^) per leaf of sea beet and of four sugar beet cultivars at 0.4 dS m^−1^.^.^Average values and standard error of the mean of six replications. SB, sea beet; SS1, salt-sensitive sugar beet; ST1, ST2, ST3 salt-tolerant sugar beet cultivars, general linear model, univariate ANOVA, *F*_4,66_ = 31.1; *P*< 0.001. Different letters indicate a significant difference at *P* < 0.05 based on Tukey's HSD post-hoc test.

The leaves of sugar beet cultivars are thinner than those of their ancestor sea beet at all salinity levels (Fig. 1G), but their area per leaf is larger than that of sea beet (*F*_4,66_ = 31.1; *P* < 0.001). Leaf area per (oldest) leaf for sea beet at 0.4 dS m^−1^ was 23 cm^2^. For cultivar SS1 it was 51 % higher; for ST1 22 % higher (not significant); for ST2 260 % higher and for ST3 it was 282 % higher than for sea beet (see Fig. 2). This may imply that high succulence and increased leaf thickness represent an essential part of the salt tolerance of sea beet. Our results also show that succulence and leaf thickness were induced in the sugar beet cultivars by increased salinity (Fig. 1F and G). Likely, the thicker leaves of sea beet, and the capability of sea beet and sugar beet cultivars to further increase leaf thickness with increased salinity, contribute to their salt tolerance.

Apparently, in the relatively salt-tolerant sea beet and sugar beet plants, photosynthetic leaf area per unit of plant biomass (LAR and SLA) is reduced with salinity. This might partially be counteracted by increased leaf thickness, representing a longer path of PAR through chlorophyll-containing leaf tissue, as expressed in our study as a ULR; i.e. the biomass increase by photosynthetic CO_2_ fixation (Fig. 1E). Our result of salinity-increased ULR (biomass increase per unit leaf area) for the sugar beet cultivars and even more strongly and persistently so for sea beet suggests that photosynthetic CO_2_ uptake through stomata and fixation is not negatively affected by salinity which is in accordance with Hampe and Marschner (1982), Papp *et al.* (1983) and Rao and Terry (1989). Functionally, changes in the morphological traits such as succulence and leaf thickness in response to increased salinity could also indicate increased water-use efficiency, implying reduced stomatal water loss per unit of leaf area.

### Perspective of improved salt tolerance and saline agriculture

Our as yet unpublished field trials on salinized land in China (Zhang *et al.* 2011) indicate that increased soil salinity may hamper germination of sugar beet, although it is salt tolerant in later growing stages. Dependent on seasonal variation of precipitation, excess (monsoon) rainfall may also seriously disturb the growth of salt-tolerant, but flood-sensitive crops such as sugar beet (Drew 1997; Colmer and Flowers 2008). Based on these observations, our research will investigate salt tolerance of seed germination, as well as flood tolerance in order to expedite the development of saline agriculture together with improved salt tolerance of crops.

## Sources of Funding

J.R., P.v.B. and R.B. are employed by the Vrije Universiteit Amsterdam, The Netherlands. D.C. participated by performing research forming part of his Master's Thesis at the Department of Systems Ecology. Y.Z. and H.L. are employees of Chang ‘an Agricultural Institute, Dong Ying, Shandong, PR China. The research of B.J. is funded by Chang ‘an Agricultural Institute, Dong Ying, Shandong, and forms part of a PhD research project at the Vrije Universiteit. The research of D.K. is funded by Project 2.3.2 of the Dutch National Research Program Knowledge for Climate with co-financing of Project ZKK-1 of Zilte Kennis Kring, and the research of B.B. was made financially possible by project Saline, Perspective, Waddenfonds, The Netherlands.

## Contributions by the Authors

J.R., R.B., D.C., B.J., D.K., P.B. and B.B. all contributed to the hydroponic culture research in the greenhouse and the analyses performed. Y.Z., H.L., B.J., D.C. and J.R. supported and performed the field work in Dong Ying, Shandong in 2012 and 2013. J.R. prepared the research paper and all co-authors gave their feedback.

## Conflicts of Interest Statement

None declared.
